# Study on Fracture Characteristics of Layered Sandstone under Asymmetric Loading

**DOI:** 10.3390/ma17102328

**Published:** 2024-05-14

**Authors:** Ruiqing Hao, Yuguo Zhou, Lin Liao, Nathan Saye Teah, Wanwen Xue, Zhiling Liao

**Affiliations:** 1 Department of Underground Engineering, College of Mining Engineering, Taiyuan University of Technology, 79 Yingze West Street, Taiyuan 030024, China; 2 Shanxi Coal Import and Export Group Co., Ltd., Taiyuan 030032, China

**Keywords:** asymmetric semi-circular bending test, anisotropic sandstone, fracture growth, maximum tangential stress, maximum energy release rate, maximum strain energy density

## Abstract

In engineering practice, layered rock masses often display obvious anisotropy while deforming and failing, and the failure mode directly impacts the engineering construction stability. In this study, the fracture failure load, fracture toughness, crack deflection angle, and failure mode of a layered rock mass under different fracture modes were analyzed by utilizing improved asymmetric semi-circular disc specimens. According to the constitutive model of transversely isotropic materials, the maximum tensile stress (MTS), maximum energy release rate (MERR), and maximum strain energy density (MSED) calculation formulas were modified, and the calculation formulas of the three prediction criteria under anisotropic materials were derived. The calculation results were compared with the experimental results. The results show that the fracture toughness and crack deflection angle were significantly affected by the weak bedding plane. As a result of applying the MTS criterion, the results are closer to the experimental results, providing a solid foundation for engineering deformation, failure, and fracture analyses.

## 1. Introduction

During the evolution of crustal structures, a large number of sedimentary rocks with layered structures are formed. These rock masses may be subjected to various external forces (such as drilling, blasting, earthquakes, etc.), and a large number of internal cracks may occur. These cracks may undergo fracture instability under the influences of various factors, such as rock slope tilting, rock mass interlayer dislocation, and roof fracture during coal mining. Most of the rock pure mode I (open-type) composite fractures, pure mode II (shear-type) composite fractures, and various composite fracture combinations under various loading modes are responsible for the fracture instability of these rock masses [1,2]. At the same time, the bedding plane direction significantly influences the failure processes. To understand the evolution law of fracture cracks and micro-fracture mechanisms, it is helpful to study the pure mode I, pure mode II, and mixed mode I/II fracture characteristics of layered rock. This understanding is important for the design, safety, and maintenance of engineering structures, such as hydraulic fracturing, slope rock control, and coal mine roadway roof support design [3].

At present, for the testing of the pure mode I fracture toughness, the International Society of Rock Mechanics (ISRM) has provided four recommended methods, but for the pure mode II rock toughness measurement, only one method is recommended, which is the punching shear test under confining pressure [4,5]. However, there is currently no commonly recommended method to measure the mixed fracture toughness of rock, while many scholars have developed various test methods based on the previous tests. For example, by using the double-notched Brazilian disk test (DNBD), Bahrami [6] was able to determine the pure mode II fracture toughness values of three different rock types: granite, marble, and limestone. Chen [7] chose anisotropic marble to carry out the cracked-ring test. The mixed mode I/II fracture toughness was determined based on the failure load measurement obtained during the test.

However, the most widely used method is the improved semi-circular bending (SCB) specimen proposed by Ayatollahi et al. [8,9], named the asymmetric semi-circular bending (ASCB) specimen. By changing the relative position of the bottom loading point, the mixed fracture of an ASCB specimen can be obtained. ASCB specimens are simple in structure and easy-to-process; thus, they are used in many studies. Wu [10] carried out a series of fracture tests on ASCB specimens made of granite. The fracture propagation trajectory, fracture initiation angle, and fracture initiation site of the specimens were carefully analyzed and combined with several mixed mode fracture criteria. Zhang [11,12] used the ASCB test to study the mixed mode I/II fracture characteristics of compacted tailing soil, calculated the mode I and mode II fracture toughness values concerning the theoretical solution of classical fracture mechanics, and then gave the fracture envelopes of the mode I and mode II fracture toughness values. In terms of prediction criteria, many scholars compare the test results of ASCB specimens with different fracture criteria to verify the test result accuracy. Guo [13] and Zhao [14] used acoustic emission technology to analyze the internal damage characteristics, failure modes, and microscopic damage mechanisms of ASCB samples.

The above studies all used ASCB samples to study homogeneous materials, but, at present, most of the materials, such as coal samples, shale, and sandstone, are not homogeneous materials with anisotropic characteristics. Nejati [15,16,17] computed the fracture energy and fracture process zone by using the asymmetric semi-circular bending method to analyze the pure mode I and pure mode II fracture toughness values of anisotropic rocks and other solid materials both experimentally and theoretically. In addition, the crack torsion angle and fracture toughness experimental results were compared with three crack propagation prediction criteria (the maximum tangential stress (MTS) [18,19,20,21], maximum energy release rate (MERR) [22,23,24,25,26], and maximum strain energy density (MSED) [27,28,29,30]) to analyze their applicability. On this basis, Sakha [31] modified the three prediction criteria, and the results show that, although the modified MERR and MTS prediction results were in close agreement with the experimental results, the modified MSED criterion was also the most inaccurate model. Zhao [32] investigated the impact of the bedding angle on the fracture toughness and fracture mode of coal specimens under pure mode II loading using the ASCB method.

According to the above analysis, although ASCB specimens have been used to analyze the two fracture modes for different anisotropic materials, few studies have been conducted on both pure mode I and pure mode II mixed fractures or their fracture failure modes and crack propagation angles. Thus, under the three fracture modes, the failure law of sandstone specimens with five bedding angles was analyzed in this study using ASCB specimens. Moreover, the crack propagation angle prediction criterion was deduced and calculated by using the theoretical calculation formula of anisotropic materials, and the range of the crack propagation angle under the three modes was obtained, which provides a reference for exploring the mesomechanical properties and fracture mechanisms of anisotropic rock masses.

## 2. Sandstone Fracture Test

### 2.1. Sample Preparation

**Figure 1:**
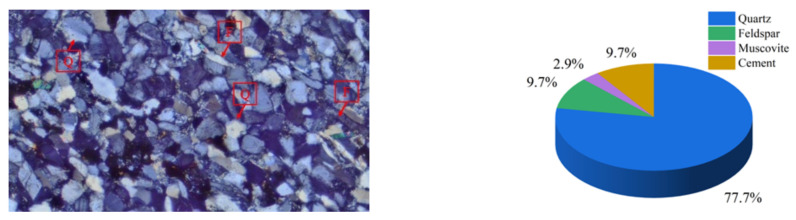
Mesostructure of rock mineral crystal (Q: quartz, F: feldspar).

The test rock samples were taken from light-brown sandstone (Changsha, Hunan, China). The rock has a light-brown color, angular appearance, and consists of particles that measure between 0.5 and 0.25 mm in size, which are well sorted and medium-rounded. There is about 90% detritus in the dump, of which 80% is quartz, 10% is feldspar, 3% is muscovite, and 10% is cement. The debris is inlaid, tightly cemented, and supported by particles. With an average density of 2.2 g/cm^3^, the rock has a block structure and parallel bedding. The sandstone contains a relatively developed bedding plane, with 1–2 mm between each bedding plane layer. Figure 1 illustrates the rock’s mineral crystal mesostructure according to the polarizing microscope analysis of the sandstone.

**Figure 2:**
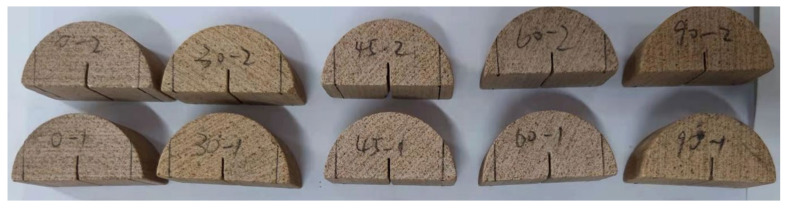
Photograph of partial samples.

Every rock sample used in the test came from large, intact blocks. The ASCB specimen’s mixed fracture strength was obtained using the ISRM fracture toughness test method by changing the relative position of the bottom loading point. The specimen’s geometry was the same as that of the SCB specimen used for testing the pure mode I fracture toughness. Five different types of SCB specimens were analyzed. The thickness of these SCB specimens is *B* = 25 mm, and the radius is *R* = 25 mm. These specimens have angles of 0, 30, 45, 60, and 90 degrees between the horizontal line and bedding plane. The specimens were polished to ensure that their end faces had no unevenness greater than 0.01 mm, and 10 mm deep, 1 mm wide grooves were cut perpendicular to the bottom centers of the samples. Figure 2 presents a photograph of the partial samples.

### 2.2. Test Scheme

**Figure 3:**
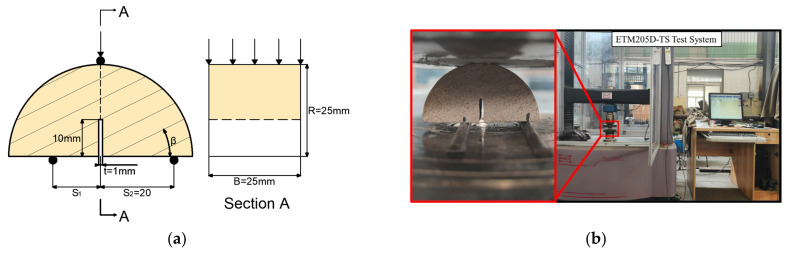
Geometric shape and testing of samples: (**a**) SCB specimens under asymmetric three-point-bending loading; (**b**) test equipment.

Figure 3 displays the specific loading method. At the bottom of the specimens are the non-fixed (*S*1) loading points and fixed (*S*2) loading points. When *S*1 = *S*2, the specimen is loaded in pure mode I. In contrast, the mixed fracture mode and pure mode II fracture modes gradually appeared on the specimens when the *S*2 loading points were fixed and the *S1* loading points approached the crack surface.

Three test groups (pure mode I fracture, mixed mode I/II fracture, and pure mode II fracture) were set up in this study based on the abovementioned ASCB test method. For SCB specimens with bedding angles of 0°, 30°, 45°, 60°, and 90° (pure mode I), a symmetrical load was applied. According to ISRM suggestions, the side point spacing (*S*) and sample radius (R) should be 0.5 ≤ *S*/*R* ≤ 0.8 for the quasi-static fracture toughness testing of materials that are similar to rocks. In this paper, *S*/*R* = 0.8; that is, *S* = 20 mm. The *S*2 loading points were symmetrically loaded when the loading mode was type I; thus, *S*2 = *S*1 = 20 mm. In the pure mode II loading, the *S1* loading point position was calculated via finite element software (Abaqus 2021), and the detailed results are shown in Section 3.3

### 2.3. Sample Loading

The ETM205D-TS microcomputer-controlled universal testing equipment (WANCE, Shenzhen, China) completed the three-point-bending loading. The displacement rate for the bottom parts of the specimens were fixed at 0.1 mm/min during the loading process to ensure stability, as shown in Figure 3b. The test continued until the specimen was destroyed after the fracture load (*P_max_*) was reached. During the test, cameras were used to record the fracture characteristics.

## 3. Test Results and Analysis

### 3.1. Load–Displacement Characteristics

**Figure 4:**
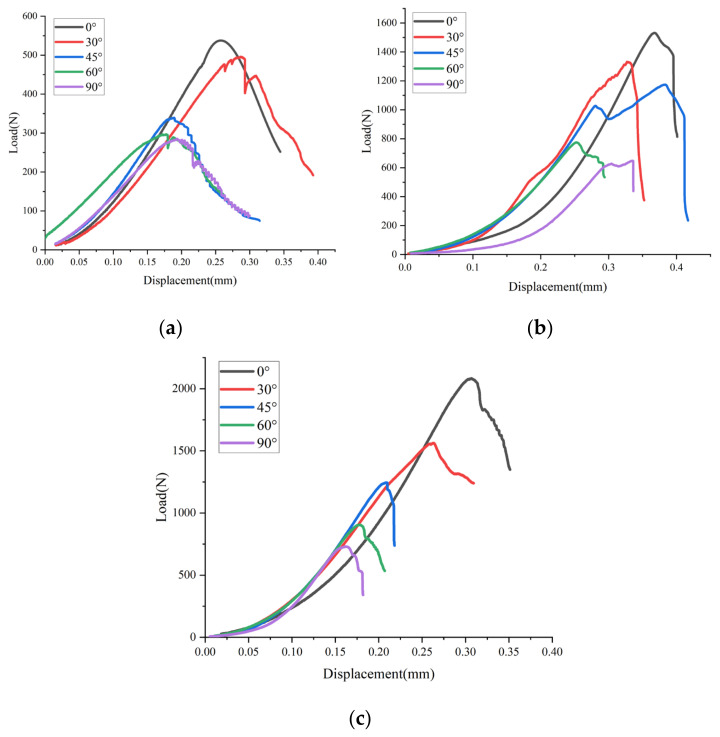
Load–displacement curves of three fracture modes: (**a**) pure mode I fracture curve; (**b**) mixed fracture curve; and (**c**) pure mode II fracture curve.

Each set of tests contained at least three samples, and their load–displacement curves were similar. To analyze the load–displacement curves, the most representative curves were selected as the analysis objects from each set of test results. The typical load–displacement curves for the three fracture modes under three-point bending are shown in Figure 4. Based on the mixed fracture mode, the load–displacement curve compaction stage had the longest displacement (around 0.15 mm) among the three curves. However, the displacements of the compaction stage under the other failure modes were less than 0.1 mm, which is because, under the mixed fracture mode, the samples were subjected to tensile and shear loads at the same time, and the internal pores and cracks were closed under the two loads. The displacement was larger, so the compaction stage was the longest. Furthermore, for the same loading mode, the elastic-stage loading rates of the specimens with five bedding angles were very close, while the loading rates under the different loading modes varied significantly. The specific average loading rates were 2153.64 N/mm (pure mode I), 6156.08 N/mm (mixed mode I/II), and 9470.20 N/mm (pure mode II), suggesting that the loading method has a significant impact on the elastic modulus, whereas the bedding angle has a minimal effect.

Moreover, four basic stages could be identified in the change in the load–displacement curves of layered sandstone under three-point-bending loading: (1) The compaction stage. The sandstone bedding specimen underwent densification as a result of the pressure-induced compression and the closure of its pores and fracture flaws. Furthermore, the displacement growth rate at this point in the curve exceeded the load growth rate. (2) The elastic deformation stage. Under the influence of an axial load, the specimen underwent elastic deformation. The load–displacement curve has a constant slope and linear positive correlation between the two. In the specimen at this time, there was a significant quantity of elastic energy. (3) The stable crack growth stage. At this stage, the load–displacement curve slope started to slow down, the specimen started to yield, and the crack kept growing along the microcrack created during the elastic deformation stage until the curve reached the peak load. At this point, the internal deformation energy of the sample could be further stored. (4) The accelerated crack propagation stage. At this stage, the load decreased substantially in the load–displacement curve. Furthermore, the elastic and deformation energies that were stored in the sample’s early stages were rapidly released as its microcracks accelerated to expand. The main macrocracks finally formed.

### 3.2. Load Variation

**Figure 5:**
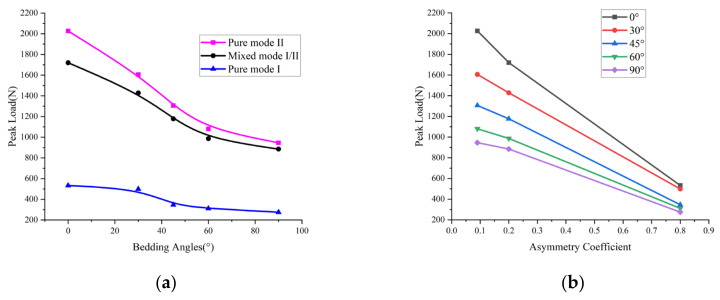
Variation law of peak load: (**a**) variation in peak load with bedding angle; (**b**) variation in peak load with asymmetry coefficient.

The peak loads obtained from the specimens’ load–displacement curves are presented in Section 3.3. In this section, we analyze the peak loads from two perspectives: (1) the relationship between the peak load and bedding angle and (2) the relationship between the peak load and asymmetry coefficient (*S*1/*R*). The peak loads of the specimens clearly showed anisotropy, as displayed in Figure 5a. Furthermore, the peak loads of the ASCB specimens decreased gradually as the bedding angle increased. The maximum value was obtained at *θ* = 0°, and the minimum value was obtained at *θ* = 90°. In addition, the diagram indicates that the peak load decreased the fastest at a bedding angle of 45°. 

Likewise, the following law may be seen in the relationship between the peak load and the asymmetry coefficient, as shown in Figure 5b: As the asymmetry coefficient increased, the specimen peak load decreased, and it reached its maximum value when the pure mode II load was applied, and it reached its minimum value when *S*1/*R* = 0.8. This was because the bearing spacing was larger when it underwent mode I loading and the specimen was subjected to a tensile load. At this time, the bearing capacity was lower. However, the bearing spacing was smaller when mode II loading was used and the specimen was subjected to a shear load. Therefore, the bearing capacities of the samples were relatively higher in these modes. In addition, as Figure 5b shows, the mode II failure peak load was about twice that of the mode I failure, which is similar to previous research findings.

### 3.3. Fracture Toughness Calculation

The stress intensity factors (*K_I_*, *K_II_*, *K_eff_*) of the ASCB specimens during crack propagation can be expressed as follows:

(1) $$K_{I}=\frac{P_{max}}{2RB}\sqrt{\pi a}Y_{I}$$

(2) $$K_{II}=\frac{P_{max}}{2RB}\sqrt{\pi a}Y_{II}$$

(3) $$K_{eff}=\sqrt{K_{I}^{2}+K_{II}^{2}}$$

where *B* is the thickness of the semi-circular specimen; *Y_I_* and *Y_II_* are the mode I and mode II dimensionless geometric factors, respectively; *P_max_* is the peak load of the load–displacement curve of the specimen. By substituting the peak load and the geometric parameters of the specimen into Equations (1) and (2), respectively, the mode I and II fracture toughness values (*K_IC_* and *K_IIC_*) can be obtained.

**Figure 6:**
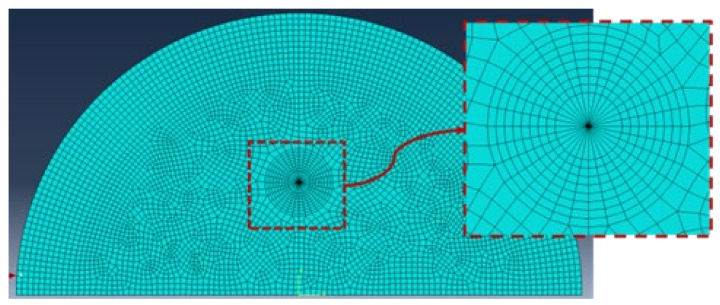
Typical SCB specimen finite element mesh.

The dimensionless geometric factors (*Y_I_* and *Y_II_*) in the formula above could not be calculated numerically because of the complicated geometry of the SCB specimens; therefore, the finite element method was used to solve the majority of these factors. In this research, to obtain the dimensionless geometric factors (*Y_I_* and *Y_II_*), we utilized the method proposed by M.R. Ayatollahi et al. [9], which is based on finite element calculation. The specimen’s geometrical properties and loading parameters were specified as follows in the simulation calculation: *R* = 25 mm, *t* = 2.5 mm, *P* = 1000 N, and *a* = 10 mm. The *S1* points were set to 20, 17.5, 15, 12.5, 10, 7.5, 5, 2.5, and 2 mm, and the *S2* points were fixed at 20 mm. The geometric factors of nine loading positions under five bedding angles were simulated to determine the *S1* point positions when pure mode II fracture occurred. The material properties were set to the real parameters of the rock sample, as shown in Table 1 [33]. Figure 6 presents a typical SCB finite element mesh. The sensitivity analysis of the local grid at the crack tip was carried out. Considering the square root singularity of the crack tip, a total of 32 triangular singular elements were set in the first ring of the element around the crack tip. The ring part was an eight-node quadrilateral grid, with a total of 256 elements. For the remaining part of the grid, a grid size of 0.05 mm was determined. To ensure the solution accuracy, the grid was set to an eight-node quadrilateral grid, and the final number of grids was 7066. The calculated geometric factor (*Y_I_* and *Y_II_*) results are shown in Table 2. Finally, the fracture toughness values of the specimens under different loading situations were obtained by substituting the computed geometric factors and peak loads obtained from the tests into Equations (1) and (2). Table 2 shows the calculation results.

### 3.4. Effect of Bedding Angle and Asymmetry Coefficient on Fracture Toughness

**Figure 7:**
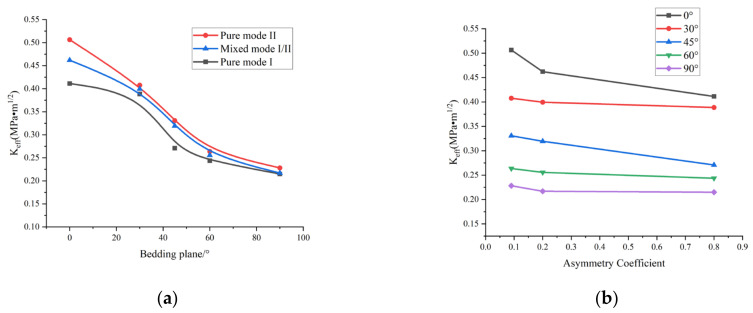
Variations in fracture toughness: (**a**) variations in fracture toughness with bedding angle; (**b**) variations in fracture toughness with asymmetry coefficient.

Figure 7 shows the pure mode I, pure mode II, and mixed mode I/II fracture toughness test results. The pure mode I fracture toughness (*K_IC_*) and pure mode II fracture toughness (*K_IIC_*) were significantly influenced by the bedding angle and asymmetry coefficient. The fracture toughness values under the three fracture conditions gradually decreased as the bedding angle increased, as seen in Figure 7a. Pure mode II had the highest fracture toughness, whereas pure mode I had the lowest. As demonstrated in Figure 7b, under different asymmetry coefficients, the fracture toughness values at different bedding angles gradually decreased as the asymmetry coefficient increased. Furthermore, while the pure mode I fracture toughness was comparable to the composite fracture toughness, the pure mode II fracture toughness was generally higher than the other fracture toughness forms.

### 3.5. Fitting Prediction Formula

According to the above data analysis, there was a relationship between the bedding angle, asymmetry coefficient, and fracture toughness. Based on the three-dimensional change diagram, the experimental results were fitted, and the formula with a good fitting degree was obtained as follows:

(4) $$\begin{array}{l} f(\alpha ,S1/R)=0.5226-0.04782\alpha -0.2734(S1/R)-0.3762\alpha^{2}+0.2152\alpha (S1/R)\\ +\;0.168{\left(S1/R\right)}^{2}+0.1839\alpha^{3}-0.04771\alpha^{2}(S1/R)-0.08177\alpha {\left(S1/R\right)}^{2} \end{array}$$

In the above formula, *α* is the bedding angle, which adopts the radian, and *S1/R* is the asymmetry coefficient. The initiation angle can be obtained by substituting the relevant parameters into the above formula.

**Figure 8:**
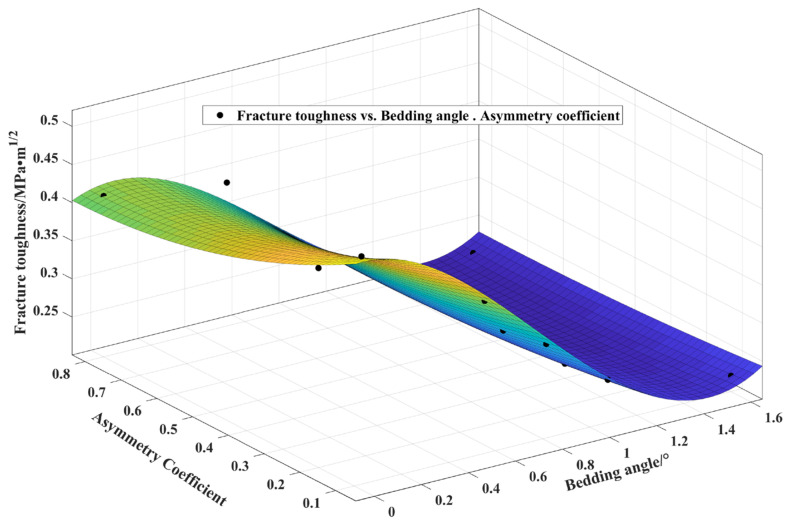
Three-dimensional view of the relationship between the bedding angle, asymmetry coefficient, and fracture toughness. (Points are test results and surfaces are fitting results).

The experimental results and the 3D image of the above expression are displayed in Figure 8, showing consistent results. The diagram shows that as the bedding angle and asymmetry coefficient rose, the fracture toughness decreased. Moreover, the impact of the bedding angle on the fracture toughness increased with the increase in the asymmetry coefficient.

## 4. Crack Instability Failure Analysis

### 4.1. Crack Initiation Angle Test Results

**Figure 9:**
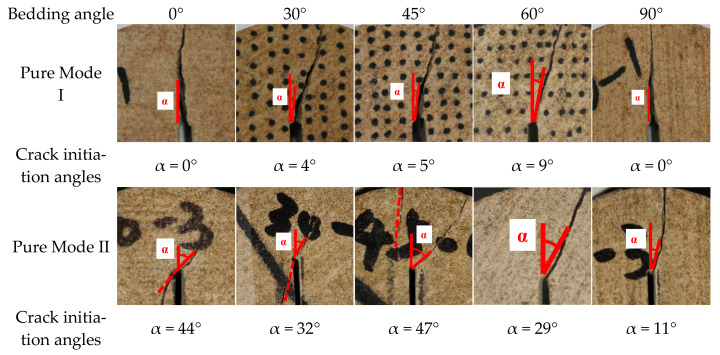

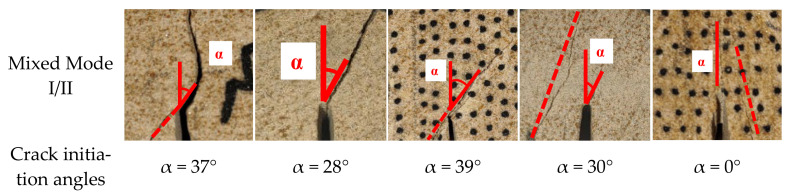
The crack initiation angles of the three fracture modes. (Points are test results and surfaces are fitting results).

The pure mode I, pure mode II, and mixed mode I/II fractures determined the crack initiation angles, as shown in Figure 9. The distribution of the initiation angles for the mode I fracture cracks, which was affected by the bedding angle, was 0°~9°; the mode II fracture cracks were distributed between 11° and 47°; and the mixed fracture cracks were distributed between 0° and 39°. These results suggest that the bedding angle plays an important role in the development of rock cracks and cannot be ignored.

Figure 9 clearly shows that the fracture propagation angles increased with the loading mode, increasing from 0° in pure mode I to 44° in pure mode II when the bedding angle was 0°. While the deflection angle of the pure mode I fracture was quite small, the specimen crack initiation deflection angles in the pure mode II and mixed failure modes were almost 28° when the bedding angle was 30°. Due to the influence of the 45° weak bedding plane, the crack propagation angles of the pure mode II and mixed fractures of the samples were about 45°. Both tended to deflect toward the loading point, and the weak bedding plane impact was more obvious; thus, there was no obvious deflection in the end. The pure mode I fracture, however, only presented a 5° deflection angle when weak bedding planes were involved. When the bedding angles were 60° and 90°, the crack propagation angles of the three failure modes were in the range of 9°~30° and 0°~11°, respectively, which were similar to those of the 45° weak bedding plane sample. This was primarily caused by the weak bedding plane; the crack was initiated along the 60° and 90° weak bedding plane directions, with no obvious deflection.

Based on the above analysis, the pure mode I fracture crack was initiated along the notch direction (that is, along the center line of the specimen). Then, because of the bedding impact, the crack propagation had a slight deviation, but there was no obvious variation in the deflection angles, which ranged from 0° to 9°. The mode II and mixed fracture cracks were initiated at a certain angle to the upper right of the notch, and the crack initiation angle was significantly increased compared with the mode I fracture. Compared to the mixed fracture, the mode II fracture had a larger crack initiation angle. The author believes that this is because the pure mode II fracture was a shear failure. In pure shear failure, because the asymmetric loading point (*S*1) is closer to the slit, it is affected by the shear effect; thus, the crack will have a larger deflection angle, which is similar to previous research results [34,35,36].

### 4.2. Theoretical Analysis of Crack Initiation Angle

At present, although there are a variety of crack instability propagation criteria, the most commonly used are still the MTS, MERR, and MSED. However, the above three criteria are all based on the calculation formulas of isotropic materials, and prediction calculations under anisotropic conditions are rarely considered. Accordingly, to obtain a modified prediction formula suitable for the crack propagation criteria of transversely isotropic materials, the three previously mentioned criteria are applied to the constitutive model of transversely isotropic materials. To verify the accuracy of this modified prediction formula, the following experiments are carried out:

(1) The maximum tensile stress (MTS) criterion

In fracture mechanics, the crack initiation angle of a pure mode I crack is 0°, and the crack initiation angle of a pure mode II crack is calculated according to the MTS criterion, as in Equation (5) [37]:

(5) $$\theta_{c}=-arccos\frac{1}{3}\approx -70.5{}^{\circ}$$

However, the above formula is only applicable to the calculation of the deflection angles of isotropic materials, while there is no unified calculation formula for anisotropic materials. Therefore, according to the MTS criterion, in this research, we deduced the crack initiation angle calculation formula for transversely isotropic materials. In the anisotropic plane problem, the polar coordinate components of the stress near the crack tip of mode I and mode II are as follows [16]:

Pure mode I:

(6) σr=KI2πrReμ1μ1 − μ2sinθ − μ2cosθ2cosθ + μ2sinθ−μ2μ1 − μ2sinθ − μ1cosθ2cosθ + μ1sinθσθ=KI2πrReμ1μ1 − μ2cosθ+μ2sinθ3/2−μ2μ1 − μ2cosθ+μ1sinθ3/2τrθ=KI2πrReμ1μ1 − μ2sinθ−μ2cosθcosθ+μ2sinθ−μ2μ1 − μ2sinθ−μ1cosθcosθ+μ1sinθ

Pure mode II:

(7) σr=KII2πrRe1μ1 − μ2sinθ − μ2cosθ2cosθ + μ2sinθ−sinθ − μ1cosθ2cosθ + μ1sinθσθ=KII2πrRe1μ1 − μ2cosθ+μ2sinθ3/2−cosθ+μ1sinθ3/2τrθ=KII2πrRe1μ1 − μ2sinθ−μ2cosθcosθ+μ2sinθ−sinθ−μ1cosθcosθ+μ1sinθ

In the formula, *r* and *θ* are the polar coordinates, *μ_1_* and *μ_2_* are the roots of the characteristic equation, and Re represents the real part of the complex number. The second formula in Equations (6) and (7) is added, and a derivative of *θ* is taken. Let (∂*σ_θ_*)/∂*θ* = 0; in this case, the formula is simplified, and the following is obtained:

(8) ∂σθ∂θ=3212πrRe1μ1−μ2KIμ1μ2cosθ−sinθcosθ+μ2sinθ−μ2μ1cosθ−sinθcosθ+μ1sinθ+ KIIμ2cosθ−sinθcosθ+μ2sinθ−μ1cosθ−sinθcosθ+μ1sinθ=0

Because $1/\left(b_{1}-b_{2}\right)=0$, there is no practical significance, and the crack initiation and propagation direction of the composite fractures of transversely isotropic materials should satisfy the following:

(9) KIReμ1μ2cosθ0−sinθ0cosθ0+μ2sinθ0−μ2μ1cosθ0−sinθ0cosθ0+μ1sinθ0+ KIIReμ2cosθ0−sinθ0cosθ0+μ2sinθ0−μ1cosθ0−sinθ0cosθ0+μ1sinθ0=0

By solving the above formula, the crack initiation angle under this prediction criterion can be calculated;

(2) The maximum strain energy density (MSED) criterion

Considering the two-dimensional case of a crack, the crack is in a composite stress field. Let the Cartesian coordinate system be (*x*, *y*), where the *x*-axis is orthogonal to the crack front and the *y*-axis is perpendicular to the crack plane. In the anisotropic plane, the stress field at the crack tip is as follows:

(10)   σxσyτxy=KI2πrReμ1μ2μ1 − μ2μ2cosθ + μ2sinθ−μ1cosθ + μ1sinθ1μ1 − μ2μ1cosθ + μ2sinθ−μ2cosθ + μ1sinθμ1μ2μ1 − μ21cosθ + μ1sinθ−1cosθ + μ2sinθ+ KII2πrRe1μ1 − μ2μ22cosθ + μ2sinθ−μ12cosθ + μ1sinθ1μ1 − μ21cosθ + μ2sinθ−1cosθ + μ1sinθ1μ1 − μ2μ1cosθ + μ1sinθ−μ2cosθ + μ2sinθ

In the formula, *r* and *θ* are the polar coordinates, *μ_1_* and *μ*_2_ are the roots of the characteristic equation, and Re represents the real part of the complex number.

The strain potential energy of the whole elastomer is as follows:

(11) $$\begin{array}{l} U=∭udxdy\\ u=\frac{1}{2}(\sigma_{x}\varepsilon_{x}+\sigma_{y}\varepsilon_{y}+\tau_{xy}\gamma_{xy}) \end{array}$$

According to Hooke’s law, the stress–strain relationship of the plane stress problem of transversely isotropic materials is as follows [15]:

(12) $$\left\{\begin{array}{l} \varepsilon_{x}\\ \varepsilon_{y}\\ \gamma_{xy} \end{array}\right\}=\left[\begin{array}{ccc} S_{11} & S_{12} & 0\\ S_{12} & S_{22} & 0\\ 0 & 0 & S_{66} \end{array}\right]\left\{\begin{array}{l} \sigma_{x}\\ \sigma_{y}\\ \tau_{xy} \end{array}\right\}$$

In the above formula, *S*_11_ = 1/*E*_1_, *S*_22_ = 1/*E*_2_, *S*_66_ = 2(1 + *ν*_12_)/*E*_1_, and *S*_12_ = −*ν*_12_/*E*_1_ = −*ν*_21_/*E*_2_.

By substituting Equation (11) into Equation (12), the formula can be simplified as follows:

(13) $$u=\frac{1}{2}S_{11}\sigma_{x}^{2}+S_{12}\sigma_{x}\sigma_{y}+\frac{1}{2}S_{22}\sigma_{y}^{2}+\frac{1}{2}S_{66}\tau_{xy}^{2}$$

Then, by substituting Equation (12) into Equation (13), the following formula can be obtained:

(14) u=14πrS11Re1μ1 − μ22KI2μ1μ22M2+2KIKIIμ1μ2MN+KII2N2+ 12πrS12Re1μ1 − μ22KI2μ1μ2MQ+KIKIIμ1μ2MW+NQ+KII2Q2+ 14πrS22Re1μ1 − μ22KI2Q2+2KIKIIQW+KII2W2+ 14πrS66Re1μ1 − μ22KI2μ1μ22W2−2KIKIIμ1μ2WM+KII2M2

In the above formula,

M=Reμ2cosθ + μ2sinθ−μ1cosθ + μ1sinθ,N=Reμ22cosθ + μ2sinθ−μ12cosθ + μ1sinθQ=Reμ1cosθ + μ2sinθ−μ2cosθ + μ1sinθ,W=Re1cosθ + μ2sinθ−1cosθ + μ1sinθ

Therefore, Equation (14) can be reduced to the following:

(15) u=1rRea11KI2+a12KIKII+a22KII2

In the above formula,

a11=12πRe1μ1 − μ22S112μ1μ22M2+S12μ1μ2MQ+S222Q2+S662μ1μ22W2a12=12πRe1μ1 − μ22S11μ1μ2MN+S12μ1μ2MW+S12NQ+S22QW−S66μ1μ2WMa22=12πRe1μ1 − μ22S112N2+S222W2+S12Q2+S662M2

Therefore, $S=a_{11}K_{I}^{2}+a_{12}K_{I}K_{II}+a_{22}K_{II}^{2}$.

According to the strain energy density factor criterion, let $\frac{\partial S}{\partial \theta }=0,\frac{\partial^{2}S}{\partial \theta^{2}}<0$, and because neither *K_I_* nor *K_II_* contains the angle *θ*, we have

(16) $$\frac{\partial S}{\partial \theta }=\frac{\partial a_{11}}{\partial \theta }K_{I}^{2}+\frac{\partial a_{12}}{\partial \theta }K_{I}K_{II}+\frac{\partial a_{22}}{\partial \theta }K_{II}^{2}=0,\;\frac{\partial^{2}S}{\partial \theta^{2}}=\frac{\partial^{2}a_{11}}{\partial \theta^{2}}K_{I}^{2}+\frac{\partial^{2}a_{12}}{\partial \theta^{2}}K_{I}K_{II}+\frac{\partial^{2}a_{22}}{\partial \theta^{2}}K_{II}^{2}<0$$

The specific calculation formula is shown in Appendix A. By solving the above formula, the crack initiation angle under the prediction criterion can be calculated;

(3) The maximum energy release rate (MERR) criterion

**Figure 10:**
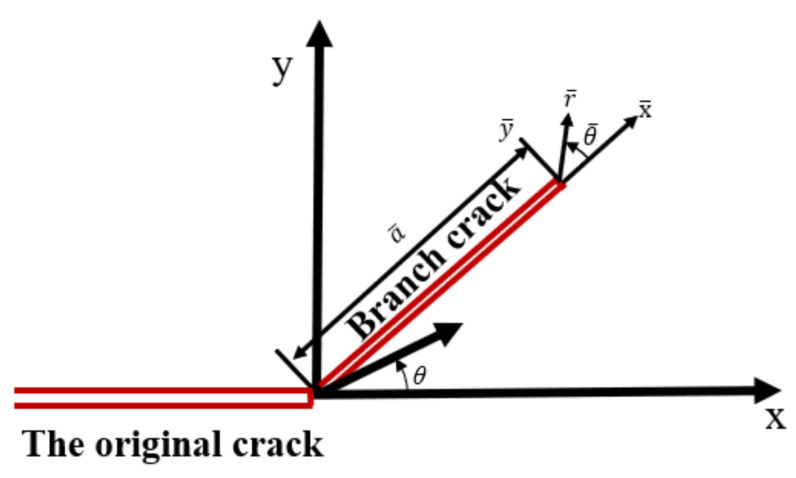
Branch crack and its related coordinate system.

The MERR criterion considers that when the composite fracture propagates, the crack propagation direction is the direction that can produce the maximum energy release rate. In this direction, when the energy release rate reaches the critical value, the crack begins to expand unstably. Using the continuity assumption, the direction of the maximum energy release rate is the direction of the maximum circumferential stress. After the composite crack expansion, new branched cracks are generated. The crack and its related coordinate system are shown in Figure 10. The polar coordinate component of the original straight crack tip stress is expressed by Equation (16). It is assumed that a branch crack of $\bar{a}$ length is generated along the *θ* = *θ_0_* direction. In order to distinguish it from the original crack, a short overline is added to the symbol of each crack. Therefore, the polar coordinate component expression of the stress at the branch crack tip is obtained by replacing *r*, *θ*, *K_I_*, and *K_II_* in Equation (17) with $\bar{r}$, $\bar{\theta }$, ${\bar{K}}_{I}$, and ${\bar{K}}_{II}$, respectively [38,39].

In the plane stress state, the energy release rate at which the crack propagates along its plane is as follows:

(17) Gmix=−πKI22S22Imμ1+μ2μ1μ2+πKII22S11Imμ1+μ2

Similarly, the energy release rate of branch cracks is as follows:

(18) G¯mix=−πK¯I22S22Imμ1+μ2μ1μ2+πK¯II22S11Imμ1+μ2

Let the crack size $\bar{a}$ approach zero, and suppose that the stress at the branch crack tip is close to the stress field at the tip of the original crack before the expansion begins; that is,

(19) $$\left\{\begin{array}{l} \underset{\bar{a}\to 0}{\lim}\bar{\sigma_{y}}{\left.\;=\;\sigma_{\theta }\right|}_{\theta =\theta_{0}}\\ \underset{\bar{a}\to 0}{\lim}\bar{\tau_{xy}}{\left.\;=\;\tau_{r\theta }\right|}_{\theta =\theta_{0}} \end{array}\right.$$

By substituting Equations (6) and (7) into the above equation, we obtain the following:

(20) K¯I0=lima¯→0K¯I=KIReμ1μ1 − μ2b3/2−μ2μ1 − μ2a3/2+KIIRe1μ1 − μ2b3/2−a3/2K¯II0=lima¯→0K¯II=KIReμ1μ1 − μ2db−μ2μ1 − μ2ca+KIIRe1μ1 − μ2db−ca

In the above formula, $\begin{array}{l} a=\left(cos\theta_{0}+\mu_{1}sin\theta_{0}\right);\;b=\left(cos\theta_{0}+\mu_{2}sin\theta_{0}\right)\\ c=\left(sin\theta_{0}-\mu_{1}cos\theta_{0}\right);\;d=\left(sin\theta_{0}-\mu_{2}cos\theta_{0}\right) \end{array}$

In this way, the energy release rate at which the branch crack begins to expand from the original crack direction along the *θ* = *θ_0_* direction is as follows:

(21) G¯mix0=−πK¯I022S22Imμ1+μ2μ1μ2+πK¯II022S11Imμ1+μ2

Therefore, according to the maximum energy release rate criterion, the crack propagation direction should satisfy the following equation:

(22) ∂G¯mix0∂θ0=−πK¯I0∂K¯I0∂θ0S22Imμ1+μ2μ1μ2+πK¯II0∂K¯II0∂θ0S11Imμ1+μ2=0

By solving the above formula, the crack initiation angle under this prediction criterion can be calculated.

### 4.3. Theoretical Calculation Results of Crack Initiation Angle

(1) Initiation angle trend analysis

**Figure 11:**
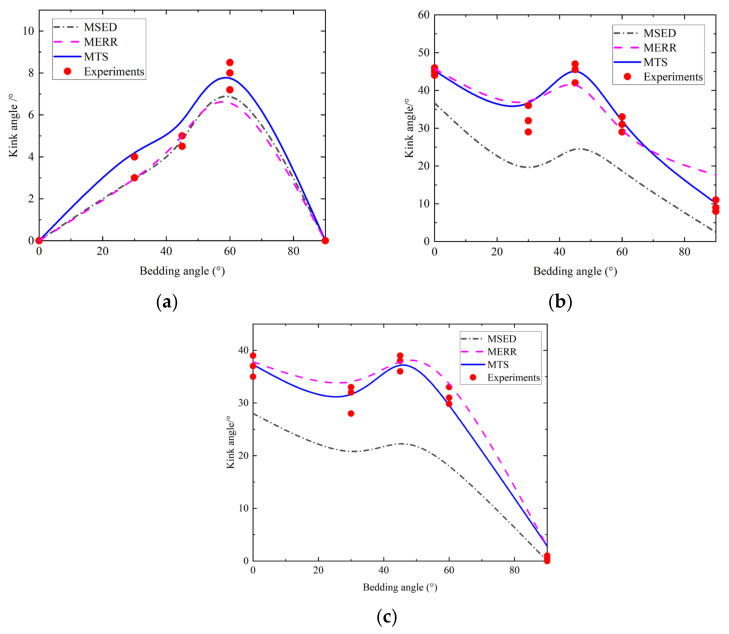
Test and calculation results of crack initiation angles under three fracture modes: (**a**) pure mode I; (**b**) pure mode II; and (**c**) mixed mode I/II.

Figure 11 displays the fracture initiation angle calculation results and tendencies. The crack initiation angle ranged from 0° to 8° when pure mode I fracture occurred. When the bedding angle ranged from 0° to 60°, the weak bedding plane had an increasing effect on the fracture initiation angle. The maximum fracture initiation angle deviation occurred around 60°, after which it gradually decreased. Both the fracture initiation angle and the bedding angle were 0° when they were 90° and 0°, respectively. Theoretically, the crack initiation angle for pure mode II fracture ranged between 2° and 47°, and the sample fracture initiation angle was significantly influenced by the weak bedding plane, as can be observed. The fracture initiation angles all expanded along the weak bedding plane when the bedding angles were 45° and 60° due to the impact of a weak bedding plane. Moreover, the fracture initiation angles were larger than those of the other bedding angles when the bedding angles were 0° and 45°. When the bedding angle was between 0° and 30°, the tendency of the crack initiation angle gradually decreased and then increased from 30° to 45° and then gradually declined again. Finally, the fracture initiation angle reached its minimal value at a 90° bedding angle. In addition, the theoretically calculated crack initiation angles in the case of mixed mode fracture ranged from 0 to 41°, and the tendency was similar to that of the pure mode II fracture.

As mentioned above, the theoretical calculations of the three fracture modes were the same as the test results within a certain range. It can be seen from the above curves that under the three fracture modes, the crack initiation angles were affected by the weak bedding plane, especially for bedding angles greater than 30°. At this time, increasing external loads were applied to the weak bedding plane as a result of the angle between the loading direction and the gradual decrease on the surface. It is more likely that cracks will expand along a weak bedding plane because of its weak mechanical characteristics. Therefore, the crack initiation angle was significantly affected by the weak bedding plane.

(2) Comparison of three prediction criteria

As shown in Figure 11, the three prediction criteria could well predict the tendency of the crack initiation angle change with the bedding angle. For the pure mode I fracture, there is not much of a difference between the predicted angles of the three fracture modes and the experimental results. However, only the MTS and MERR criterion prediction findings are close to the experimental angles in the cases of pure mode II and mixed fractures. Although the MSED criterion prediction results are the same as the experimental change tendencies, there is a certain gap between the values. Specifically, the errors between the predicted results of the three fracture modes and the test results are shown in Table 3. The average MTS criterion errors are in the range of 3.25~4.54%, and the errors are small. The MERR criterion error range is from 8.77% to 12.58%, while the MSED criterion had large errors of 10.85~34.72%. The research results are similar to those of previous research. Therefore, the MTS can be considered the most accurate and simple criterion, and it is more suitable for crack initiation angle prediction in practical engineering.

(3) Fitting prediction formula

According to the above data analysis, there is a relationship between the bedding angle, asymmetry coefficient, and crack initiation angle. Following the fitting of the experimental results based on the three-dimensional change diagram, the following formula with a good fitting degree was obtained:

(23) $$\begin{array}{l} f(\alpha ,S1/R)=44.06-21.28\alpha -23.88(S1/R)+49.01(\alpha^{2})\\ -\;53.16\theta (S1/R)-38.29\alpha^{2}-30.34\alpha^{3}+14.79\alpha^{2}(S1/R)+65.96\alpha {\left(S1/R\right)}^{2} \end{array}$$

In the above formula, *α* is the bedding angle, which adopts the radian, and *S*1/*R* is the asymmetry coefficient. The initiation angle can be obtained by incorporating the relevant parameters into the above formula.

**Figure 12:**
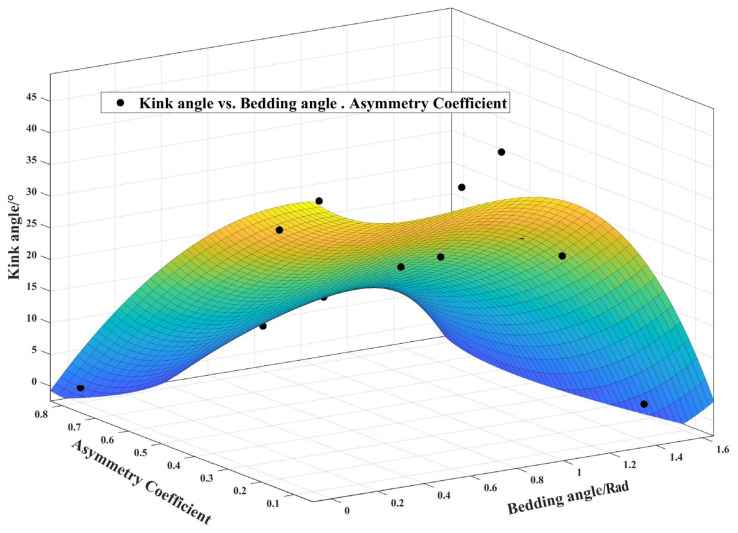
Three-dimensional view of relationship between bedding angle, asymmetry coefficient, and crack initiation angle. (Points are test results and surfaces are fitting results).

The above 3D image expression and the experimental results, which show good consistency, are displayed in Figure 12. The diagram shows that when the bedding angle and asymmetry coefficient rose, the crack’s initial angle decreased. Furthermore, the bedding angle had a greater effect on the fracture initiation angle as the asymmetry coefficient increased.

### 4.4. Effect of Bedding Angle on Failure Mode

**Figure 13:**
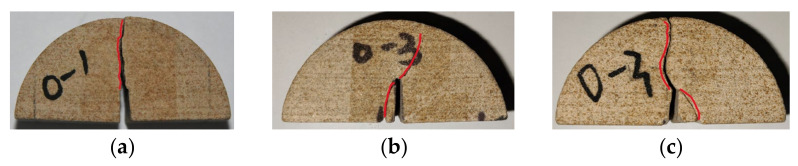
Three failure modes when the bedding angle was 0°: (**a**) pure mode I; (**b**) pure mode II; and (**c**) mixed mode I/II. (The red line represents the crack path).

As shown in Figure 13, the fracture propagated in the slit direction near the loading location with pure mode I loading and a bedding angle of 0°. Moreover, secondary cracks were rare during the propagation process, and the crack surface was rough. When pure mode II loading developed, the crack initially expanded at a 44° angle away from the slit. When a certain path was extended along this direction, it deflected and continued to deflect toward the loading point to form a curved path. However, under the condition of mixed loading, due to the mixed fracture shear and tensile forms, the crack first expanded in the 37° direction during the expansion process and then reached a certain layer. Then, the surface fracture initiated a deflection towards the loading location, and, finally, it deflected twice and penetrated. Moreover, the sandstone in this test was brittle, and the strength was low, which led to a high stress concentration degree at the bottom support point. Therefore, during the mode II and mixed loading processes, there was damage at the bottom support point, which follows the findings of a previous study [40,41].

**Figure 14:**
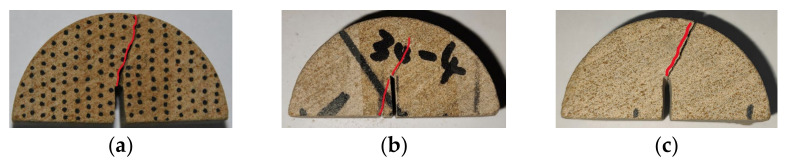
Three failure modes when the bedding angle was 30°: (**a**) pure mode I; (**b**) pure mode II; and (**c**) mixed mode I/II. (The red line represents the crack path).

For pure mode I failure, the crack propagated along the 4° direction when the bedding angle was 30°, as shown in Figure 14. Although the crack was affected by the bedding angle, it did not completely propagate along the direction of the weak bedding plane. At this stage, the fracture mostly showed a rough surface due to tensile failure. In terms of pure mode II failure, the same crack expanded from the end of the slit along the 32° direction due to the influence of the bedding angle. At this time, the crack was mainly shear failure; thus, the crack surface was relatively smooth and had a tendency to deflect toward the loading point. However, due to the influence of the weak bedding plane, the expansion trend was not as obvious as when the bedding angle was 0°. Under the mixed loading condition, the crack propagated from the end of the slit along the 28° direction. At this time, the crack was affected by the bedding angle but did not propagate completely toward the bedding angle direction. Additionally, when the fracture grew, it was slightly more likely to deflect toward the loading point; even so, it finally expanded to the right side of the loading point.

**Figure 15:**
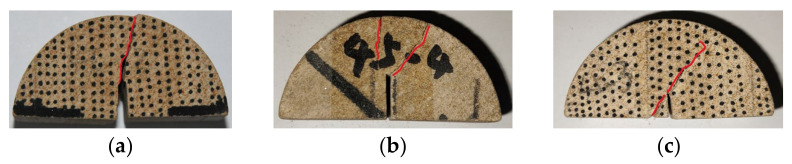
Three failure modes when the bedding angle was 45°: (**a**) pure mode I; (**b**) pure mode II; and (**c**) mixed mode I/II. (The red line represents the crack path).

When the bedding angle was 45°, as shown in Figure 15, the crack had an obvious tendency to expand along the weak bedding plane. At this stage, the weak bedding plane gradually affected the crack propagation path due to the 45° angle that resulted from the loading direction and bedding plane. For pure mode I, the crack propagation angle was smaller than those of the other two deflection angles, which is because the failure mode at pure mode I was tensile failure, and the tensile failure strengths of the matrix and weak bedding plane were small. In this failure mode, it was easy for the crack to penetrate the matrix and for the bedding plane to produce a failure path with a small deflection angle. When a pure mode II fracture occurred, the crack was mainly affected by the weak bedding plane; thus, the crack propagated directly from the end of the slit along the 47° direction. At this time, the crack was mainly shear failure; thus, its surface was relatively smooth. However, a fracture closest to the S1 loading point occurred as a result of the stress concentration at the upper loading point. When a mixed fracture happened, damage occurred at the lowest loading point as the same crack expanded in the 39° direction. At this time, the crack was rougher than in pure mode II.

**Figure 16:**
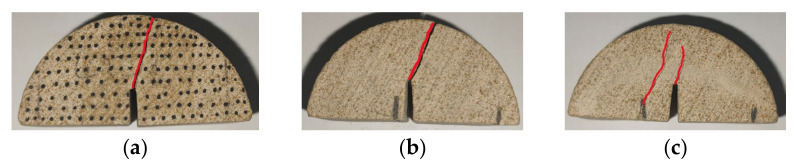
Three failure modes when the bedding angle was 60°: (**a**) pure mode I; (**b**) pure mode II; and (**c**) mixed mode I/II. (The red line represents the crack path).

The loading direction and the weak bedding plane had an angle of 30° when the bedding angle was 60°. The weak bedding plane at this point mainly impacted the crack initiation and growth. Tensile failure was the primary reason for the pure mode I failure, as shown in Figure 16. The fracture expanded in the 9° direction due to the weak bedding plane effect. When a certain path was extended, the crack continued to expand in the 60° direction. At this time, the weak bedding plane was stretched, destroyed, and finally connected. Shear failure occurred on the weak bedding plane between the two beds when the pure mode II failure was influenced by it. The fracture initiated and expanded in the 29° direction. Due to the impact of the weak bedding plane, the crack was inclined toward the loading point, although, at this point, there was no obvious deflection, and the surface of the fracture was relatively smooth. In the mixed loading, the crack propagated in the 30° direction. When it extended to a certain path, it began to deflect towards the loading point. At this time, due to the high stress concentration at the top loading point, another crack from the weak bedding plane to the bottom loading point direction appeared, but it was not completely connected.

**Figure 17:**
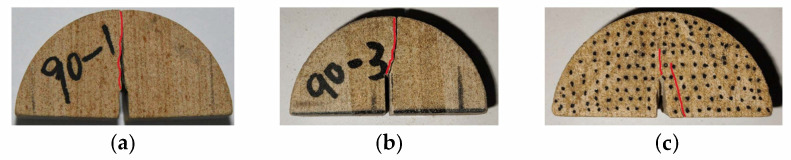
Three failure modes when the bedding angle was 90°: (**a**) pure mode I; (**b**) pure mode II; and (**c**) mixed mode I/II. (The red line represents the crack path).

As shown in Figure 17, the crack propagation path expanded over the weak plane layer in the slit direction when the bedding angle was 90°. The loading direction was parallel to the direction of the weak bedding plane. When pure mode II failure occurred, the crack had a smaller deflection angle and finally penetrated along the weak plane layer. The three failure modes were mainly affected by the weak bedding plane. The pure mode I failure was caused by the interface tension between a certain weak bedding plane; thus, the crack surface was rough. However, the pure mode II failure destroyed the shear failure of the weak plane layer, and the crack surface was relatively smooth. While the mixed type produced tensile and shear failure at the same time, the crack surface was relatively smooth.

According to the research above, the crack propagation paths of the three failure modes were significantly influenced by the bedding angle. When the bedding angle was 45°–90°, the effect was the most obvious. The other bedding angles had little influence on the failure form because of the large angle that existed between the loading direction and weak bedding plane. Under the three failure modes, the pure mode I fracture was mainly tensile failure; thus, the crack surface was rough. Moreover, the crack deflection angles at the five bedding angles were small, and stress concentration and failure did not occur at the loading point. Under the pure shear failure mode, the crack path presented a curve form with a deflection towards the loading point when the bedding angle was small. However, when the bedding angle was greater than 45°, it was significantly affected by the weak bedding plane, and the deflection trend was not obvious. Shear failure occurred directly on the weak bedding plane between the two substrates, and, due to the high stress concentration degree at the bottom loading point (S1) and the close distance from the slit, a crack towards the end of the slit was generated at the loading point. Furthermore, the crack surfaces of all the mode II fractures were relatively smooth. Similarly, under mixed loading conditions, the crack was also affected by the weak bedding plane, and secondary cracks appeared in the direction of the loading point.

## 5. Discussion

Based on the results of this research, the author offers the following opinions as a reference for related research:

(1) Because the peak load and fracture toughness decreased gradually with the increase in the bedding angle, the author believes that, under the action of external load, the pure type II fracture failure was pure shear failure. Compared with the pure tensile failure form of pure mode I, the sandstone samples selected in this experiment were brittle materials, and their tensile strengths were weaker than their shear strengths. Therefore, the pure mode II fracture toughness and peak load were higher than those of pure type I. Similarly, under the influence of the weak bedding surface, the loading direction was perpendicular to the weak surface of the bedding at 0°, whether it was subjected to pure tensile failure or pure shear failure. Moreover, it was difficult to extend the crack along the slit direction. Only after resisting the larger load did the crack begin to expand. When the bedding angle ranged from 30° to 60°, the crack began to expand along the direction of the weak bedding plane, and the orientation of the bedding plane became the main factor in its failure. At 90°, the weak bedding plane was parallel to the loading direction, and pure tensile failure occurred between the bedding weak planes. Similarly, pure shear failure occurred along a certain weak bedding plane. At this time, it was easier to generate crack propagation along the slit direction. Therefore, the fracture toughness and peak load were the largest at 0° and the smallest at 90°.

(2) Because the influences of parameters such as the tensile stress were not considered, some of the test results have certain deviations from the theoretical calculations. However, the calculation result range is the same as that of the test results; thus, the theoretical calculations in this research are reasonable. In engineering practice, when the angle between the direction of the external load and the weak bedding plane is small, the failure mode or crack initiation of the layered rock mass will be greatly affected by the bedding angle. Therefore, the influence of a weak bedding plane must be taken into account, whether selecting a support mode, excavating a tunnel, or using other engineering practices.

## 6. Conclusions

In this research, the brittle fracture test and a theoretical analysis of layered sandstone specimens under asymmetric loading were carried out. First, finite element software (Abaqus 2021) was used to compute the loading points of the different fracture modes based on the preparation of the SCB specimens with different bedding angles. Using an asymmetric three-point-bending fracture test, the peak load, fracture toughness, and crack initiation angle of a rock mass with different bedding angles were calculated. Then, based on the theoretical calculation of transverse isotropic materials, the prediction formulas of the MTS, MERR, and MSED crack propagation criteria were modified, and the calculation results were compared with the experimental values. The conclusions are as follows:

(1) The sample load–displacement curves can be broadly categorized into four stages under the three fracture modes: compaction, elastic deformation, stable crack growth, and accelerated crack growth. The loading method has a greater influence on the layered sandstone elastic modulus than the bedding angle.

(2) The peak load and fracture toughness indicated similar variations, both of which decreased gradually as the bedding angle increased. The pure mode II fracture load and fracture toughness were the greatest, while the pure mode I fracture load and toughness were the smallest. The sample fracture failure was significantly impacted by the weak bedding plane. Likewise, as the asymmetry coefficient increased, so did the peak load and fracture toughness.

(3) The crack initiation angles calculated via the three modified criteria had the same variation law as the bedding angle of the rock sample. Moreover, the specific crack initiation angles calculated using the MTS and MERR criteria are close to the experimental results, while the MSED criterion prediction results are quite different from the experimental values. Among the three modified criteria, the MTS criterion had the smallest error between the calculated results and test results, and its calculation formula is the simplest and can be used as a better reference criterion in engineering applications.

(4) The influence of the asymmetry coefficient and bedding angle on the crack initiation angle were analyzed, and a fitting formula between the three is given, which has a good fitting degree. The initiation angle decreased with the increases in the bedding angle and asymmetry coefficient. Moreover, with the increase in the asymmetry coefficient, the influence of the bedding angle on the crack initiation angle was greater.

(5) An analysis and summary are provided for studies on the fracture failure mechanisms of rock samples with different bedding angles. The crack propagation paths of the three failure mechanisms were impacted by the bedding angle. The greatest obvious impact occurred when the bedding angle was between 45° and 90°.

## Figures and Tables

**Table 1:** Basic mechanical parameters of sandstone.

Bedding Angle /°	Compressive Strength /MPa	Elastic Modulus /GPa	Poisson’s Ratio	Tensile Strength /MPa
0	53.36	6.46	0.21	2.65
30	39.97	3.03	0.22	2.46
45	21.65	1.75	0.20	2.33
60	34.48	2.89	0.24	1.86
90	41.47	3.15	0.28	1.25

**Table 2:** Sandstone fracture toughness test results.

Bedding Angle /°	Asymmetry Coefficient	*Y_I_*	*Y_II_*	Average Peak Load/N	*K_I_* /MPa·m^1/2^	*K_II_* /MPa·m^1/2^	*K_eff_* /MPa·m^1/2^
0	0.8 (Pure mode I)	5.46	0.00	533.33	0.41	0.00	0.41
	0.066 (Pure mode II)	0.00	1.76	2026.75	0.00	0.51	0.51
	0.2 (Mixed mode I/II)	1.61	1.00	1720.08	0.39	0.24	0.46
30	0.8 (Pure mode I)	5.46	0.62	499.08	0.39	0.00	0.39
	0.086 (Pure mode II)	0.00	1.79	1505.50	0.00	0.38	0.41
	0.2 (Mixed mode I/II)	1.39	1.40	1293.58	0.26	0.26	0.40
45	0.8 (Pure mode I)	5.48	0.59	346.67	0.27	0.00	0.27
	0.09 (Pure mode II)	0.00	1.79	1305.75	0.00	0.33	0.33
	0.2 (Mixed mode I/II)	1.30	1.40	1177.25	0.22	0.23	0.32
60	0.8 (Pure mode I)	5.50	0.43	311.42	0.24	0.00	0.24
	0.09 (Pure mode II)	0.00	1.72	940.00	0.00	0.23	0.26
	0.2 (Mixed mode I/II)	1.25	1.33	786.58	0.14	0.15	0.25
90	0.8 (Pure mode I)	5.51	0.00	275.30	0.21	0.00	0.21
	0.1 (Pure mode II)	0.00	1.70	736.25	0.00	0.18	0.23
	0.2 (Mixed mode I/II)	1.24	1.21	604.75	0.11	0.10	0.22

**Table 3:** Error percentages between test results and theoretical results.

Bedding Angle /°	MSED			MTS			MERR		
	Pure Mode I	Pure Mode II	Mixed Mode I/II	Pure Mode I	Pure Mode II	Mixed Mode I/II	Pure Mode I	Pure Mode II	Mixed Mode I/II
0	0.00	16.69	24.32	0.00	2.79	0.69	0.00	3.89	2.16
30	25.98	39.18	34.20	9.57	7.95	2.74	24.46	0.16	12.21
45	15.57	38.72	38.48	1.96	1.81	6.22	5.43	12.98	1.54
60	12.68	37.93	35.96	4.71	2.28	0.87	13.99	13.10	24.02
90	0.00	41.09	0.00	0.00	7.86	9.11	0.00	32.74	5.71
Mean discrepancy	10.85	34.72	26.59	3.25	4.54	3.93	8.77	12.58	9.13

## Data Availability

Data are contained within the article.

## Appendix A

(1) The MSED criterion is as follows:

(A1) $$S=a_{11}K_{I}^{2}+a_{12}K_{I}K_{II}+a_{22}K_{II}^{2}$$

Therefore, the calculation formula of the MSED criterion is as follows:

(A2) $$\frac{\partial S}{\partial \theta }=\frac{\partial a_{11}}{\partial \theta }K_{I}^{2}+\frac{\partial a_{12}}{\partial \theta }K_{I}K_{II}+\frac{\partial a_{22}}{\partial \theta }K_{II}^{2}=0$$

In the above equation,

(A3) ∂a11∂θ=Re1μ1 − μ22μ1μ22πS112μ1μ22 + S222μ1μ2+  S12μ12 + μ222 + S662μ1μ2acosθ − sinθcosθ  +  bsinθcosθ  +  asinθ3+  1μ1 − μ22μ1μ22πS112μ1μ22 + S222μ1μ2+  S12μ12 + μ222  +  S662μ1μ2bcosθ − sinθcosθ  +  bsinθ3cosθ  +  asinθ− 12π1μ1 − μ22S112μ12μ24 + S222μ12+  S662μ1μ22 + S12μ1μ22bcosθ − sinθcosθ  +  bsinθ2− 12π1μ1 − μ22S222μ22 + S112μ1μ22μ12+  S662μ1μ22 + S12μ1μ22acosθ − sinθcosθ  +  asinθ2∂a12∂θ=12πRe1μ1 − μ22S11μ12μ23 + μ13μ222 + S12μ12μ2 + μ1μ222+ S12μ13 + μ232 + S22μ1 + μ22− S66μ1μ2μ12μ2 + μ1μ222acosθ − sinθcosθ + bsinθcosθ + asinθ3+ bcosθ − sinθcosθ + bsinθ3cosθ + asinθ− 12π1μ1 − μ22S11μ1μ24 + S12μ1μ22 + S12μ1μ22 + S22μ1 − S66μ1μ22bcosθ − sinθcosθ + bsinθ2− 12π1μ1 − μ22S22μ2 + S11μ14μ2 + S12μ12μ2 + S12μ12μ2 − S66μ12μ2acosθ − sinθcosθ + asinθ2∂a22∂θ=12πRe1μ1 − μ22S112μ12μ22 + S12μ1μ2+ S662μ1μ2 + S222acosθ − sinθcosθ + bsinθcosθ + asinθ3+ 12π1μ1 − μ22S112μ12μ22 + S12μ1μ2+ S662μ1μ2 + S222bcosθ − sinθcosθ + bsinθ3cosθ + asinθ− 12π1μ1 − μ22S112μ24 + S12μ12+ S222 + S662μ22bcosθ − sinθcosθ + bsinθ2− 12π1μ1 − μ22S12μ22 + S662μ12+ S112μ14 + S222acosθ − sinθcosθ + asinθ2.

(2) The MERR criterion

According to the MERR criterion, the crack propagation direction satisfies the following equation:

(A4) ∂G¯mix0∂θ0= −πK¯I0∂K¯I0∂θ0S22Imμ1 + μ2μ1μ2 + πK¯II0∂K¯II0∂θ0S11Imμ1 + μ2=0

In the above equation,

(A5) ∂K¯II0∂θ0=KIReμ1μ1 − μ2b3/2 − 12μ1μ1 − μ2D − a3/2 − 12C − KIIRe121μ1 − μ2D + C

(A6) ∂K¯I0∂θ0=32KIReμ1μ1 − μ2D − μ2μ1 − μ2C + 32KIIRe1μ1 − μ2D − C

In the above equation, C=Reμ1cosθ0 − sinθ0D=Reμ2cosθ0 − sinθ0

Therefore,

(A7) K¯I0∂K¯I0∂θ0=KI2Reμ1μ1 − μ22b3 − 12μ1μ1 − μ22b3/2D − μ1μ1 − μ2ab3/2 − 12μ1μ1 − μ2b3/2C− μ1μ1 − μ22ab3/2 + 12μ1μ2μ1 − μ22a3/2D + μ2μ1 − μ2a3 + 12μ2μ1 − μ2a3/2C+ KIKIIReμ1μ1 − μ22b3 − ab3/2 − 12Db3/2 − a3/2 − 12D + Cμ1 − μ22μ1b3/2 − μ2a3/2− 1μ1 − μ2ab3/2 − a3 + 12Cb3/2 − a3/2− 12KII2Re1μ1 − μ22b3/2 − a3/2D + C

(A8) K¯II0∂K¯II0∂θ0=KI2Reμ1μ1 − μ22db2 − 12dbD − μ1μ1 − μ2a3/2db + 12dbC+ μ1μ2μ1 − μ2212caD − b3/2ca + μ2μ1 − μ2a2c + 12caC+ KIKIIReμ1μ1 − μ22db2 − cb3/2a − 12Ddb − ca− 1μ1 − μ2da3/2b − ca2 + 12db − caC − 12D + Cμ1 − μ22μ1db − μ2ca− KII2Re121μ1 − μ22D + Cdb − ca
